# Preliminary evaluation of a school-based resilience-promoting intervention in a high-risk population: Application of an exploratory two-cohort treatment/control design

**DOI:** 10.1371/journal.pone.0177191

**Published:** 2017-05-08

**Authors:** Michael Pluess, Ilona Boniwell, Kate Hefferon, Aneta Tunariu

**Affiliations:** 1 Queen Mary University of London, London, United Kingdom; 2 Anglia Ruskin University, Cambridge, United Kingdom; 3 University of East London, London, United Kingdom; Stellenbosch University, SOUTH AFRICA

## Abstract

Applying innovative methodology, we explored the efficacy of *SPARK Resilience Programme*––a new universal school-based resilience-promoting programme––regarding effects on depression symptoms and resilience in a high risk population in England. Quantitative and qualitative methods were combined in an exploratory two cohort treatment/control design with one cohort serving as the control group (single assessment) and a subsequent cohort as the treatment group (assessed before and immediately after treatment as well as 6 and 12 months after treatment ended), involving a total of 438 11–13 year old girls, According to analyses, depression symptoms were significantly lower directly after treatment and at 6 months but no longer at 12 months. Resilience scores, on the other hand, were significantly higher in the treatment cohort compared to the year-ahead control cohort at post-treatment and both follow-up assessments. Qualitative results demonstrated beneficial teacher experience overall. The current study provides first evidence for the efficacy of *SPARK Resilience Programme*. Furthermore, the applied two cohort treatment/control mixed methods design proved helpful for the preliminary testing of a school-based universal intervention programme efficacy in an authentic setting.

## Introduction

Child and adolescent mental health is increasingly at risk in Western societies [1–3]. According to a relatively recent study in England––comparing two representative cohort studies separated by 20 years––the prevalence of emotional problems has been rising, especially among females [4].

Adolescent mental health problems––specifically depressive disorders––have been associated with numerous negative outcomes, including academic and interpersonal difficulties, physical health problems, smoking, substance abuse, and suicide [5–7] with depressive disorders being more prevalent in girls and in children growing up in economically deprived neighbourhoods [8,9].

Given the deleterious effects of depression and the observation of increases in adolescent mental health problems in many Western countries it is not surprising that there have been multiple efforts aimed at the promotion of emotional well-being and resilience in children as a means of combating mental health problems before they develop [10,11]. Most of these efforts have the same goal of equipping children––especially those at risk for mental health problems––with adaptive coping skills and related competencies in order to increase their resilience to life stress and future challenges. Although resilience refers to a process rather than a trait, generally defined as resistance to the negative effects of stressful experiences or the ability to quickly recover (i.e. “bounce back”) from them [12], several individual characteristics have been associated with observed resilience in empirical work, including positive self-perception, self-efficacy, optimism, self-control, problem solving skills, and sense of meaning [13]. Consequently, many preventative programmes aim at the promotion and consolidation of such resilience-related individual attributes.

According to several meta-analyses, interventions specifically developed to prevent depression through the promotion of resilience and protective factors have been generally found to be effective (e.g., [14,15–17]). The most recent meta-analysis included 81 studies with a tot sample of 31,794 participants [18]. The effect sizes for interventions aimed at reducing depression symptoms were relatively small with *g* = .23 at post-intervention, *g* = .12 at 6–12 months after the intervention, and *g* = .11 more than 12 months after the intervention. Although treatment effects on depression symptoms tend to be relatively small according to the different meta-analyses (*r* = .11 to .24) they generally remain significant at follow-up assessments with larger effects found for targeted (i.e., higher risk) compared to universal samples, samples with higher proportions of females, programmes delivered by professional therapists compared to trained teachers, and for shorter compared to longer programmes (for a detailed report on moderating variables, see [16]). However, the majority of the existing evaluation studies of resilience promoting programmes focused exclusively on the effects interventions had on depression symptoms rather than on resilience-related traits in spite of the rational of most applied interventions to prevent depression through the promotion of resilience and associated competencies (but see [17,19]).

The current study overcomes limitations of existing work by investigating the effects of a new resilience-promoting intervention on depression symptoms as well as resilience-related attitudes, behaviours, and personality traits, such as a positive self-concept, emotion regulation, adaptive stress coping strategies, finding meaning, building on strengths, and self-efficacy. The *SPARK Resilience Programme* is a universal school-based resilience-promoting intervention programme specifically developed for 10–12 year old children from deprived urban communities in England [20].

The empirical evaluation of universal school-based programmes is inherently challenging [21]: 1) treatment effects are generally moderate to small [17] which requires rather large samples for both treatment and control groups; 2) randomized allocation into control and treatment groups is often problematic due to participants being nested within classes which are further nested within schools with both classes and schools often significantly varying on outcome measures at baseline; and, 3) evaluations that include separate treatment and control schools depend on the compliance of many parties involved (i.e. school administration, teachers, students, parents). Consequently, randomized controlled trials for the evaluation of universal school-based intervention programmes are often difficult, time-consuming, and expensive.

Testing preventative programmes under authentic “real-life” conditions is of great importance when evaluating whether a programme might be suitable and beneficial for children, especially those from more economically disadvantaged backgrounds. Not surprisingly, the administration of universal school-based teacher-presented interventions in at-risk populations faces additional challenges that are likely to undermine the detection of treatment effects: missing data due to lack of compliance and cooperation by children and their parents, frequent school absence of the children, and overburdened teachers at understaffed schools to mention just a few of the typical obstacles. Given these challenges, we developed and applied an alternative approach to test the SPARK resilience programme in an exploratory fashion, addressing many of the aforementioned methodological difficulties while permitting a preliminary evaluation of SPARK in an authentic setting in spite of limited resources. Importantly, this approach should be understood as an additional methodological tool for the preliminary evaluation of interventions in authentic settings rather than an alternative approach to existing and established study designs.

The current evaluation involved a sample of 438 11–13 year old girls at a state school in one of the most deprived neighbourhoods of England representing the population most at risk for depressive disorders [8,9]. For example, whereas the 1 year prevalence rate for emotional problems in 11–16 year old girls from financially well off households (i.e., more than £770 weekly income) was 3.7% in the United Kingdom in 2004, this rate increased up to 12.9% for girls from families with low income (i.e., less than £100 per week) in the same year [22]. In comparison, in the same study boys had a prevalence rate of 1.5% in high income families and 11.5% in low income families.

The programme was delivered by trained teachers in the regular class setting as part of the standard curriculum. In order to address the typical methodological challenges of studies aimed at evaluating universal school-based programmes we combined a two cohort treatment/control design with a mixed methods approach [23]. The intervention was conducted in the treatment cohort only, which included all children in the same year at the same school, while the complete year-ahead cohort of the same school served as control group. Besides comparing quantitative data between treatment and control cohorts, qualitative methods (i.e., focus group with teachers) were applied in order to collect important additional information regarding the implementation of the intervention.

In summary, the current study is advancing the field of preventative interventions by investigating the effects of a resilience-promoting intervention programme on both adaptive and maladaptive outcomes in a high-risk population under real-life conditions applying an exploratory innovative study design.

## Materials and methods

### Procedure

**Fig 1:**
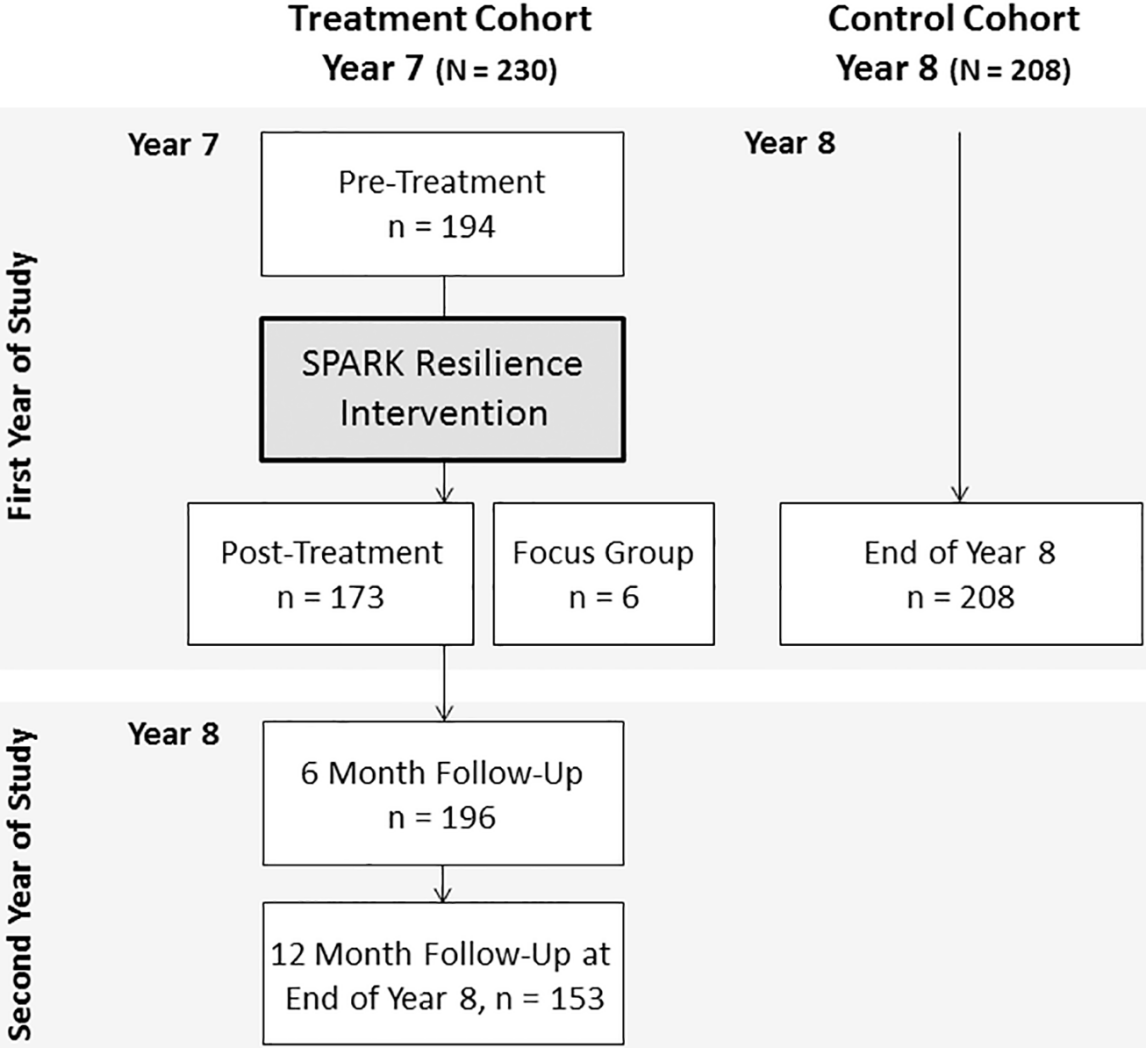
Flow chart of the applied two cohort treatment/control design.

The current study combines both quantitative and qualitative methods (i.e. mixed methods) in order to evaluate the SPARK resilience programme [23]. For the quantitative component of the study we applied a two cohort treatment/control design with one cohort serving as the control group and a subsequent cohort as the treatment group. The SPARK resilience programme was delivered to all children of the same cohort in year 7 (i.e. 6^th^ grade) as part of the official curriculum at a girls-only comprehensive state funded school in East London, United Kingdom, in place of the standard Personal, Social, Health, Economic education curriculum (PSHE) which was delivered to the year-ahead control cohort. Quantitative data was collected on laptop computers during class at school, using an online questionnaire service, immediately before and after delivery of the programme as well as 6 and 12 months after the programme was completed. In order to understand the intricacies of implementing the SPARK programme, including what components worked well or not so well, a single focus group was conducted with teachers, shortly after completion of the intervention. The complete year-ahead cohort served as a control group assessed only once at the end of school year 8, exactly one year before the 12-month follow-up assessment of the treatment cohort was conducted. Consequently, the control cohort assessment corresponds to the 12-month follow-up assessment of the treatment group with girls in control as well as treatment cohort both approaching the end of year 8 but separated by one year (see Fig 1 for flow chart).

The study received ethical approval from the University of East London research ethics committee. Parents were informed per letter about the evaluation component of the resilience curriculum and given the opportunity to exclude their child from participation instead of providing written informed consent for study participation. However, none of the parents chose to opt out of the evaluation study. Each participating child was informed at the initial assessment by their teacher that they have the right to stop filling in questionnaires at any time without giving a reason and without any negative consequences. The ethics committee approved this consent procedure.

### Participants

The study included a total of 438 11–13 year old girls of which 230 were in the treatment (eight classes ranging in size from 26–30 girls) and 208 in the year-ahead control cohort (eight classes ranging in size from 24–30 girls). Due to attrition, failure to complete all questionnaires in time, and absence from school when data collection took place, sample sizes vary across measures and assessments with 186–194 (81–84%) girls at pre, 165–173 (72–75%) at post, 185–196 (80–85%) at 6 month, 153–158 (67–69%) at the 12 month assessment, and 177–197 (85–95%) in the control cohort. According to attrition analyses, there were no significant differences on any demographic characteristics nor depression and resilience scores between children that participated and those that didn’t at any time point of the study except at pre-assessment where response differed as a function of maternal education with mothers of non-participating children being slightly less educated (*χ2* = 10.61, *p* = .03). Importantly, the applied multi-level modelling allowed for inclusion of all 230 girls in the treatment condition into the analysis regardless of missing data. At the initial assessment girls in the treatment cohort were on average 11.4 years old (*SD* = .49 years). There was no significant difference in age at the end of year 8 between treatment cohort at 12 months follow-up (*M* = 12.9 years, *SD* = .37) and control cohort (*M* = 12.9 years, *SD* = .30). The sample was ethnically diverse with 51.7% Asian, 19.6% Mixed, 17.0% African/Caribbean, 10.0% Caucasian, and 1.7% Middle Eastern in the treatment and 43.3% Asian, 17.3% Mixed, 31.3% African/Caribbean, 6.7% Caucasian, and 1.4% Middle Eastern in the control cohort. Distributions of ethnicities in treatment and control cohorts were significantly different (*χ2* = 12.78, *p* = .01). However, there were no significant differences in family size (both group with *M* = 4.6 persons per household, *SD* = 1.81) or child reported paternal education between treatment and control cohorts (both cohorts combined: 1.8% with less than secondary school, 20.3% with only secondary school, 21.0% with a university degree, 13.0% more than one university degree, and 43.8% unknown by the child). Child reported maternal education, on the other hand, differed significantly between treatment and control group (*χ2* = 10.60, *p* = .03) with 2.2% versus 7.7% with less than secondary school, 27.0% versus 26.9% with only secondary school, 21.7% versus 16.8% with a university degree, 9.6% versus 5.8% with more than one university degree, and 39.6% versus 42.8% unknown by the child, respectively, for treatment and control group. All children attended the same school in the borough of Newham, which was ranked the third most deprived area in all of England in the 2010 index of deprivation [24].

### Intervention

The SPARK Resilience Programme is a new universal school-based positive education programme [for details of the intervention, see 20]. Developed specifically for deprived neighbourhoods, the programme builds on cognitive-behavioural therapy (see Table 1 for summary of the different CBT concepts covered throughout the programme) and positive psychology concepts (i.e. resilience, post-traumatic growth) with the explicit goal of fostering resilience and associated skills as well as preventing depression. The programme is delivered in 12 one-hour sessions across 3–4 months by local school teachers which have been trained extensively by professional psychologists over two consecutive days (i.e. weekend) and provided with all necessary teaching materials (i.e. teacher’s guidebook with detailed curriculum for each session, DVD’s with videos and slides, props, and workbooks for participating children).

**Table 1:** Active CBT components included in the SPARK resilience programme.

Lesson	Programme Elements	CBT Equivalents
1. What is resilience	Multiple resilience definitions are considered	Being in control and flexibility are emphasised
2. Let’s SPARK	SPARK acronym	CBT model
	**S**ituation	Situation
	**P**erception	Automatic negative thoughts
	**A**utopilot (affect)	Emotional reactions
	**R**eaction	Behavioural reactions
	**K**nowledge	Core beliefs reinforced
3. Parrots of perception	Metaphor of a “parrot”	Automatic negative thoughts or cognitive distortions
4. Parrots under the spotlight	Naming some of the “parrots”	Major types of cognitive distortions
	The “Blamer”	Blaming
	The “Judge”	Always being right
	The “Looser”	Mislabeling
	The “Giver Upper”	Pessimistic or negative bias
	The “Worrier”	Catastrophising
	The “Faulty”	Personalisation
	The “Whatever”	Minimisation
5. The sticky path	Metaphor of a “sticky path”	Illustration of an interaction between emotional, behavioural and physiological reactions
6. Parrot on trial	Examining the evidence for and evidence against; the trust thermometer; looking out for a confirmation bias.	Disputation; the trust thermometer; confirmation bias.
7. The jury is out	Looking for alternative explanations	Disputation, development of more flexible thinking
8. A dose of distraction	Strategies for dealing with negative affect	Exercising, breathing, relaxation strategies
9.-12.	Content based on positive psychology concepts and theory	

Organised around the *SPARK* acronym, the programme teaches children to break down their responses to stressful situations into five components: **S**ituation, **P**erception, **A**utopilot, **R**eaction and **K**nowledge. Through the use of hypothetical scenarios, children are taught how everyday *Situations*, as a function of their individual and unique *Perceptions*, tend to trigger their *Autopilot* (i.e. automatic emotional responses). Children are instructed to identify their subsequent behavioural *Reactions* and observe what *Knowledge* they gained from the experience. To help students understand these concepts, they are introduced to the “parrots of perception”––imaginary creatures representing common maladaptive cognitive distortions. The programme teaches students how to challenge their interpretation of adverse situations and consider other alternatives by putting their parrots “on trial”, understanding and modifying their automatic emotional responses, and learning to control negative behavioural reactions. Alongside, students are introduced to the skills of assertiveness and problem solving, and are helped to build their “resilience muscles” through identifying their strengths, social support networks, sources of positive emotions and reflection on previous experiences of resilience and self-efficacy. (For more detailed information on the intervention please contact the corresponding author).

### Treatment fidelity

In order to investigate treatment fidelity and children’s engagement a subset of teachers and their classes were observed by the first author during selected SPARK sessions. During these sessions it was observed (but not systematically assessed) whether teachers followed the detailed curriculum provided in the teacher’s guide while delivering the programme and whether children understood the programme and were using the SPARK work book. Generally, observed teachers appeared to adhere to the curriculum and children seemed to understand and engage with the content as reflected in their active participation during the observed sessions.

### Measures

All measures were based on child self-report and assessed with computer based questionnaires. The same questionnaire/online tool was applied at all assessment points for both treatment and control cohorts.

#### Demographics

Children reported their gender, age in years, ethnicity of mother and father, number of persons living in their household, and education of their mother and father.

#### Resilience

The Resilience Scale (RS; [25]) is a self-report questionnaire designed to measure attitudes, behaviours and personality characteristics associated with psychological resilience defined as “capacity to live a full and rewarding life”. The measure includes 25 statements (e.g., “my belief in myself gets me through hard times” and “I am determined”) that are rated on a seven-point scale ranging from “1 = strongly disagree” to “7 = strongly agree”. Higher values reflect greater resilience. Internal consistency (alpha) of RS for the treatment cohort was .92 at the pre, .95 at the post, .94 at the 6 months, .96 at the 12 months assessment, and .88 for the control cohort. Although the RS has not been developed specifically for the use with children and adolescents several reviews suggest that the scale is adequate for the measurement of resilience in adolescents [26,27].

#### Depression

Symptoms of depression were assessed with the Centre for Epidemiologic Studies Depression scale (CESD; [28]), a widely used 20-item measure inquiring about the presence of different depression symptoms in the past seven days (e.g., “I felt sad” and “I thought my life had been a failure”) on a four-point scale ranging from “1 = rarely or none of the time” to “4 = all or most of the time”. Scores can range from 0–60 with higher values reflecting more depression symptoms. Internal consistency (alpha) of CESD for the treatment cohort was .83 at the pre, .88 at the post, .88 at the 6 months, .92 at the 12 months assessment, and .88 for the control cohort. We used the adult version of the CESD which has been used with children and adolescents in previous research and performs well across ages [29]. Using a rather high cut-off point of 30 for severe depression [30,31], the prevalence rates for depression for the treatment cohort were 7.7% at the pre, 6.9% at the post, 8.7% at the 6 month, and 12.0% at the 12 month assessment. The control cohort had a depression prevalence rate of 10.7%. These depression rates based on elevated CESD scores are substantially higher than the average 1 year prevalence rate of emotional problems of 6.0% in 11–16 year old girls in Great Britain in 2004 [22] confirming the high-risk nature of the sample.

### Statistical analysis

Given the two cohort treatment/control design of the study with four repeated measures within the treatment cohort but only one assessment of the control cohort, we adopted the following statistical approach: Change across the four repeated measures *within* the treatment cohort was tested with growth curve analysis which allows inclusion of all participants that provided data at least at one of the four assessment points. Predicted values of the growth curve models with the best model fit were then used to test for differences *between* each treatment cohort assessment and the control cohort with independent sample t-tests (i.e. pre vs control, post vs control, 6M vs control, and 12M vs control). According to this approach, missing data across repeated measures is fully accounted for and preliminary empirical support for the efficacy of the intervention is provided if all of the three following conditions are met: (1) significant change over time within the treatment cohort; (2) no significant difference between pre-treatment scores and control scores; (3) significant difference between any post-treatment scores and control scores.

Exploratory data analysis included examination of variables for missing data, distribution, and outliers. Associations between demographic variables and outcome measures were evaluated using univariate analyses of variance (ANOVA) and bivariate correlations (Spearman, two-tailed). T-tests for independent samples and χ2-tests were applied to compare treatment and control cohorts on demographic variables. Cohen’s *d* was calculated as a measure of effect size based on means and standard deviations using an online calculator [32]. Effect size of within-condition change was corrected for correlation between repeated measures. The level of significance was set at α = .05. According to power analyses, the sample had sufficient power (*P* = .80) to detect effect sizes of *d* = .25 in independent t-tests (comparisons between treatment and control cohorts) and *d* = .23 in dependent t-test (comparisons between assessments within treatment cohort). All statistical analyses were carried out using SPSS version 20 for Windows.

### Qualitative study

#### Design

We administered a single focus group interview with six teachers to generate narrative data for analysis with qualitative analytic methods [33] in order to understand the experience of the SPARK resilience programme from the teachers’ perspectives.

#### Procedure

Following the 12-week intervention, teachers were sent an information package with a letter asking to take part in a focus group to get a group sense of the programme’s effect. The teachers were asked read the information sheet and sign a consent form. They were asked to respect anonymity and were briefed on the focus group process. The focus group centred on their thoughts and experiences of the SPARK Programme. A set of six questions were used to guide but not confine the discussion, which allowed participants to influence the direction of the conversation (i.e.: 1.What was your overall experience of the SPARK programme; 2.What was it like to implement?; 3.How do you feel the children received the programme?; 4.What did you like about the programme?; 5.What would you change?; 6.Do you think others would benefit from it?). At times, probes such as “Can you tell me more about…?” were used to fully understand the participant’s comments. Following the focus group, participants were offered debriefing and the chance to ask questions. All data was recorded using an mp3 player and the material was transcribed by an external transcription agency.

#### Analysis

Inductive Thematic Analysis [34] was applied to evaluate the data and map out major interpretative themes. Thematic analysis is the analysis of textual material that looks for major themes, beyond surface level description. The researcher attempts to organise the text into coherent sections. After transcription, the third and fourth authors, as well as a research assistant, read through and became familiar with the text. Once the transcripts were read and analysed for preliminary themes individually and across the group, the authors conducted a detailed, line-by-line analysis, before moving on to highlight broader overarching themes. The emerging thematic map was collectively visited and re-visited in order to compile an ever-higher level of abstraction, resulting in a final list of themes regarding teachers’ experiences of the SPARK resilience programme.

## Results–quantitative study

### Preliminary analysis

According to univariate analyses of variance outcome measures did not differ as a function of child ethnicity in neither cohort nor did depression and resilience scores differ between classes at any assessment points except depression scores in the treatment cohort at the 6 months follow-up assessment (*F*_(7,188)_ = 2.52, *p* = .02). Similarly, bivariate correlations yielded no significant association between family size and outcome measures. Paternal education, on the other hand, was significantly associated with resilience scores at pre (*r*_119_ = .28, *p* < .01) and post assessment (*r*_97_ = .24, *p* < .05) in the treatment cohort as well as with resilience scores in the control cohort (*r*_100_ = .25, *p* < .05). Maternal education was significantly associated with depression scores in the treatment group at post-assessment (*r*_103_ = -.25, *p* < .05) and the 6 months follow-up assessment (*r*_111_ = -.20, *p* < .05) but not with resilience. In the control group maternal education was unrelated to depression but significantly associated with resilience (*r*_100_ = .35, *p* < .01). However, due to the large number of children unable to report their father’s or mother’s educational backgrounds (43.5% and 39.6% in the treatment, and 44.2% and 42.8% in the control group for fathers and mothers, respectively) parental education was not included as a covariate.

Descriptive statistics and bivariate correlations of outcome measures are reported in Tables 2 and 3, separately for treatment and control cohort, respectively.

**Table 2:** Descriptive statistics and unadjusted associations for outcome variables of the treatment cohort (N = 230)

	Variables	Mean Value	Standard Deviation	Sample Size	1	2	3	4	5	6	7	8
1	Resilience Pre	120.59	25.95	186	––							
2	Resilience Post	125.92	27.31	165	.57**	––						
3	Resilience 6M	123.18	26.49	185	.47**	.50**	––					
4	Resilience 12M	123.92	27.43	153	.24**	.29**	.35**	––				
5	Depression Pre	17.53	8.28	194	-.36**	-.34**	-.28**	-.22*	––			
6	Depression Post	16.30	9.25	173	-.37**	-.54**	-.50**	-.32**	.50**	––		
7	Depression 6M	16.20	9.28	196	-.34**	-.32**	-.43**	-.29**	.38**	.69**	––	
8	Depression 12M	17.20	10.55	158	-.27**	-0.18	-.28**	-.38**	.24**	.46**	.65**	––

Note

^#^*p* < .10.

*p < .05.

**p < .01.

**Table 3:** Descriptive statistics and unadjusted associations for outcome variables of the control cohort (N = 208)

	Variables	Mean Value	Standard Deviation	Sample Size	1	2
1	Resilience	117.63	25.13	177	––	
2	Depression	18.40	9.27	197	-.29**	––

*Note*.

^#^*p* < .10.

*p < .05.

**p < .01.

### Primary analysis

For each outcome, data was fitted to three separate unconditional growth curve models. The first model included only the intercept and a linear slope, whereas the second model included also a quadratic slope and the third model a cubic slope in addition to the linear and quadratic ones. Models were then compared on a range of standard fit indices (e.g. AIC, BIC). Only the model with the best fit for each outcome is reported.

#### Resilience

**Fig 2:**
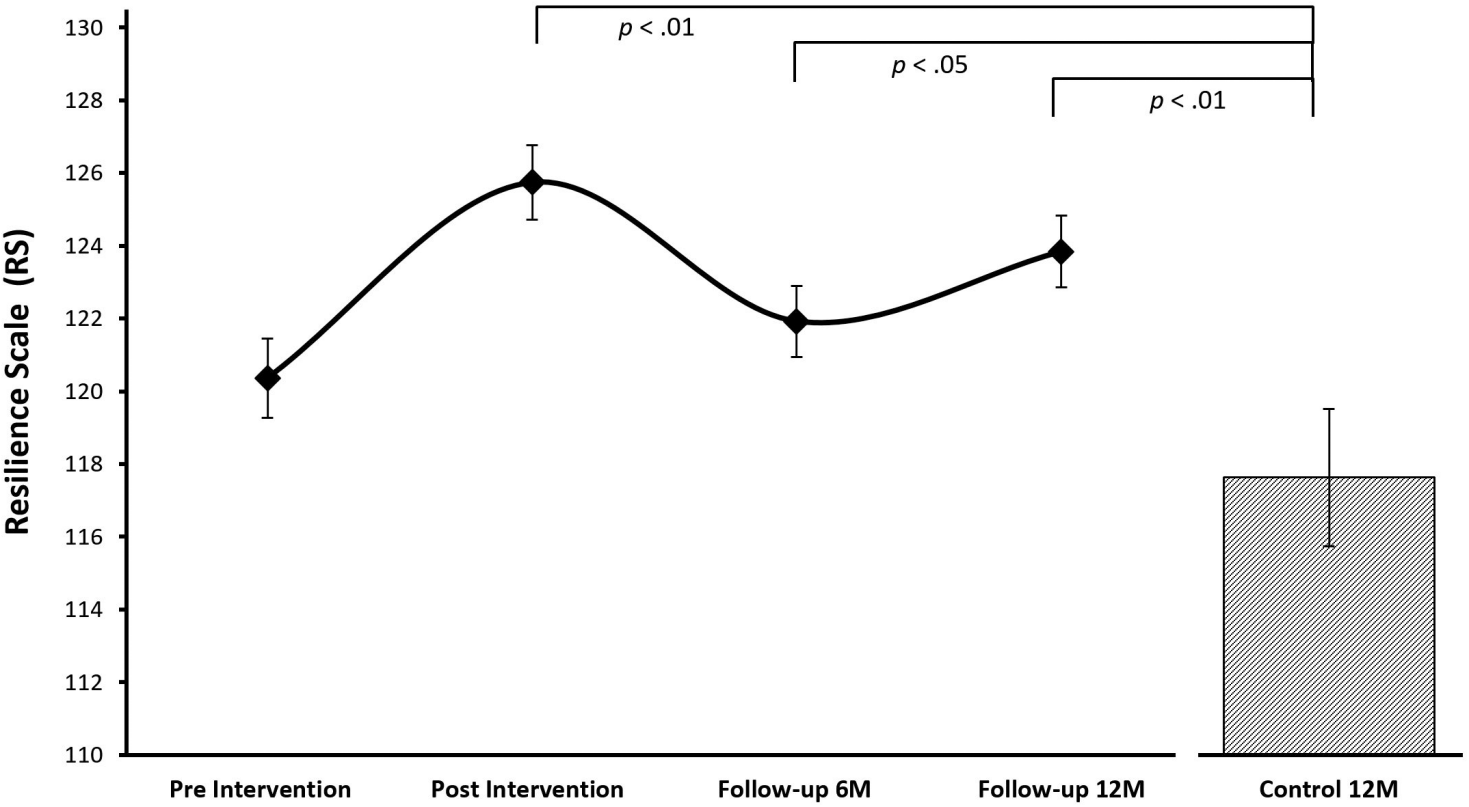
Growth curve model for resilience. Growth curve model-predicted resilience mean values for the different assessments of treatment and control cohorts (the control cohort assessment is directly comparable to the 12 month follow-up assessment of the treatment cohort).

The growth curve model including linear (*B* = 9.64, *p* = .09), quadratic (*B* = 10.20, *p* = .04), and cubic (*B* = 2.46, *p* = .03) slopes fitted the resilience scores of the treatment cohort best (see Fig 2). Follow-up dependent t-tests of model predicted resilience scores between the repeated assessments suggest that resilience scores increased from pre to post treatment (*t*_(228)_ = -25.49, *p* < .01, *d* = .31), decreased from post to 6 month follow-up assessment (*t*_(228)_ = 18.10, *p* < .01, *d* = -.25) and then again increased slightly at the 12 months follow-up assessment (*t*_(228)_ = -9.10, *p* < .01, *d* = .13). Importantly, resilience scores of the control cohort were not significantly different from the treatment group at the pre-treatment assessment (*t*_(404)_ = 1.33, *p* = .19) but significantly lower compared to the post-treatment (*t*_(404)_ = 4.00, *p* > .01, *d* = .40), 6 month follow-up (*t*_(404)_ = 2.15, *p* = .03, *d* = .22), and 12 month follow-up assessment (*t*_(404)_ = 3.10, *p* > .01, *d* = .31) suggesting that the SPARK resilience intervention led to significant and persistent growth in resilience scores in the treatment cohort (see Table 4).

**Table 4:** Summary of results based on growth curve model-predicted resilience and depression scores

Outcome	Change over time in treatment cohort (slope)	Mean differences between Treatment and Control Cohorts (independent t-test)			
		Pre	Post	6 Months	12 Months
Resilience	Linear: *B* = 9.64^#^ Quadratic: *B* = 10.20* Cubic: *B* = 2.46*	2.18	8.05**	4.28*	6.17**
Depression	Linear: *B* = 1.21 Quadratic: *B* = .47*	-.66	-1.79*	-1.98*	-1.24

*Note*.

^#^*p* < .10.

*p < .05.

**p < .01.

#### Depression

**Fig 3:**
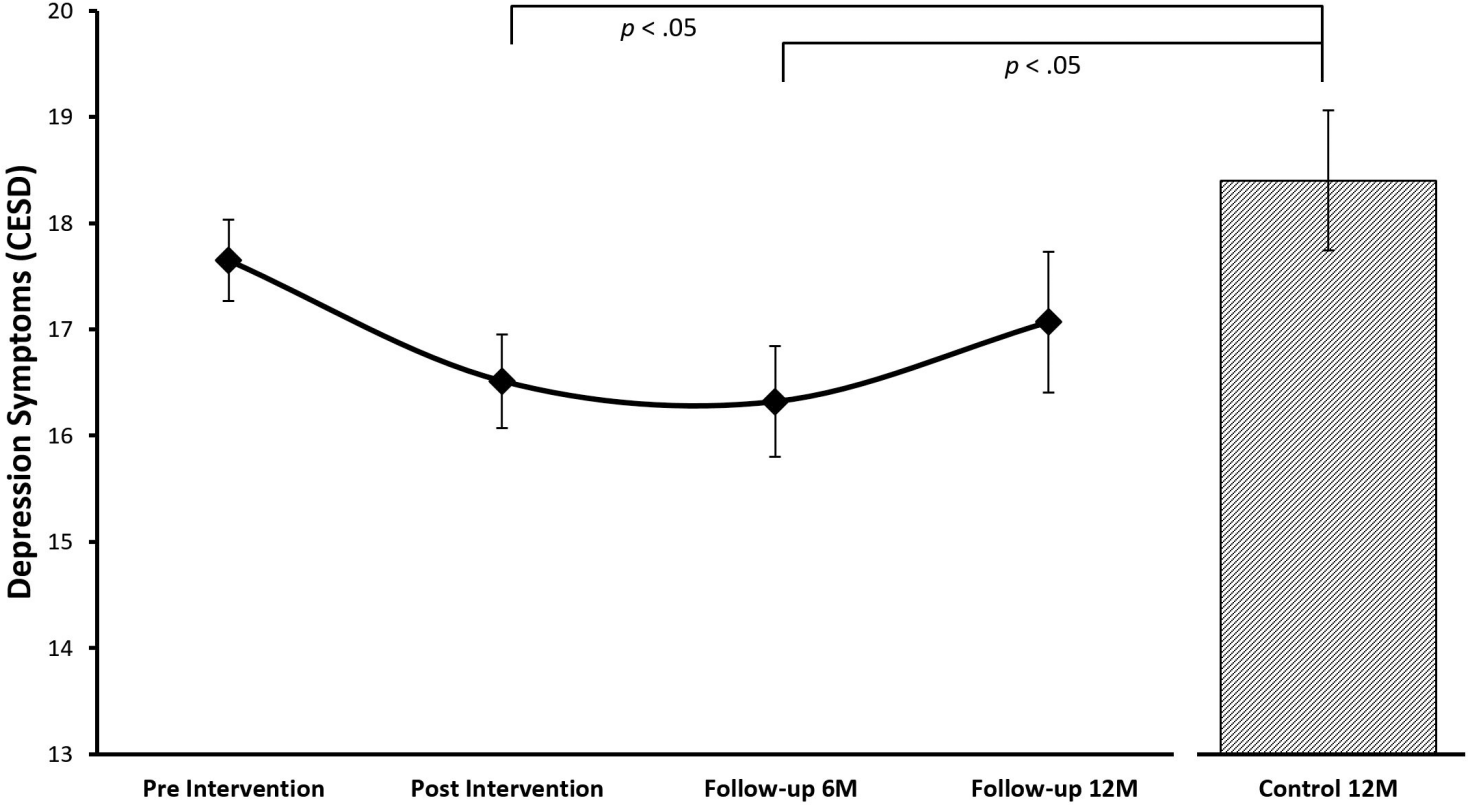
Growth curve model for depression. Growth curve model-predicted depression mean values for the different assessments of treatment and control cohorts (the control cohort assessment is directly comparable to the 12 month follow-up assessment of the treatment cohort).

The growth curve model including a linear (*B* = 1.21, *p* = .11) and quadratic (*B* = .47, *p* = .04) slope fitted the depression scores of the treatment cohort best (see Fig 3). Follow-up dependent t-tests of model predicted depression scores between the repeated assessments suggest that depression scores decreased from pre to post treatment assessment (*t*_(229)_ = 9.14, *p* > .01, *d* = -.20) but then increased again between the 6 and 12 months follow-up assessments (*t*_(229)_ = -6.02, *p* > .01, *d* = .08) so that depression scores at pre-treatment and 12 months follow-up assessments were no longer significantly different (*t*_(229)_ = 1.56, *p* = .12, *d* = .08). Depression scores of the control cohort were not significantly different from the treatment cohort at the pre-treatment assessment (*t*_(425)_ = -.91, *p* = .36) and the 12 months follow-up assessment (*t*_(425)_ = -1.49, *p* = .14) but significantly higher compared to the post-treatment (*t*_(425)_ = -2.43, *p* = .02, *d* = .24) and 6 month follow-up scores (*t*_(425)_ = -2.57, *p* = .01, *d* = .25) suggesting a depression reducing effect of the SPARK resilience intervention in the treatment cohort up to 6 months after the intervention ended but not beyond (see Table 4).

## Results–qualitative study

As previously discussed, thematic analysis of the focus group with six teachers led to the identification of three overarching main themes, indicating that the programme was experienced as a successful and beneficial intervention: (1) *Emotional Toolkit*, (2) *Empathetic Camaraderie*, and (3) *Teacher and School as Key to Success*.

### Emotional toolkit

Overall, the interviewed teachers perceived the programme as helpful in fostering important transferable life skills necessary for the development of emotional resilience. (Textual items such as ‘um, ah, erm’, etc. as well as stutters, have been removed from all presented quotes for clarity and flow. […] indicates a break in the participant’s speech).

[…] what this does, I suppose, is provide them with a toolkit, doesn’t it? A toolkit of how to respond […]

There was a strong consensus that SPARK’s pedagogy and teaching materials provided a medium for engaging with complex emotional responses in a realistic, everyday approach:

What I liked most was the chance to talk about resilience with students in a group […] to be pro-active about helping them to deal with things that they may come across in their everyday lives and issues or problems, confrontations and how do you deal with that in a better and more positive way […]

Another aspect of SPARK which emerged as salient in the teachers’ experience was the impact of the vocabulary used (e.g., perception, self-fulfilling prophecy) and, the ease with which the students accepted and incorporated the new terminology in their everyday use:

When I went on the [training course] course, I thought it was really, really interesting but I wondered how adaptable the students would be, whether they would take on board some of the ideas. My class […] really found it really interesting.

All participants commended the teacher training and guidance materials which accompanied SPARK. Together with SPARK’s pedagogical and conceptual structure, the accompanying materials enhanced teachers’ confidence and “personal ownership” which led to students’ efficient learning at an appropriate pace. Equally, the visual representations of core concepts were also deemed to be effective in translating the concepts from teacher to student:

[…] the initial lesson, the starting lesson, I thought the use of the particular clips worked really, really well and they would refer back to the term resilience throughout and I think it was because that was so visual at the beginning that they were able to really understand […]

Teachers also appreciated that pupils’ suggestions of their own SPARK examples facilitated greater understanding and ownership of the process:

I also think there’s more ownership if they’re doing that, if they’re creating their own stories […] And they’re much more involved with the whole thing which is obviously a positive, because then they can understand to more depth.

### Empathetic camaraderie

Participation in the SPARK resilience programme not only strengthened the relational communication between students and teachers, but also positively influenced the school’s general culture with a sense of empathetic camaraderie. The SPARK classroom became a forum for sharing experiences and emotions––for both teacher and students. This open environment gave the participants the permission to acknowledge and validate the universality of emotions (across age), promoting consideration of others and personal responsibility taking.

Before where I might have just said “that’s not very nice, you need to listen. That’s not very respectful”. You seem to get a lot more if I said to them, “how do you think she feels about that?” Because you’re putting them in a situation where she’s having to be resilient because of what you’re doing. And I found that that was a big impact.

SPARK was found to foster empathy and improve relationships with others in the classroom. Ultimately, pupils’ recognition and appreciation that everyone has different perspectives, different experiences, and that they, therefore, needed to develop considerations for others’ thoughts, feelings and opinions, prompted a culture shift in the school.

In order to benefit from the exercises aimed at learning empathy, the teachers believed that pupils needed a certain level of openness towards others and a readiness to shift the attentional frame of reference from self to others. Some teachers spoke of this readiness in terms of students having “a certain level of maturity” linked with chronological age:

I think it would be better in year eight or at the very end of year seven. I really did think they [y7s] were too young. […] I mean, my kids got something out of it. I think they’d have got more if we’d done it later but they did get something.

Others linked this ‘readiness’ to developmental stages. For instance, some argued that teenagers were less empathetic given a strong preoccupation with self-presentation, which may have negative effects on the efficacy of the SPARK resilience programme:

I’m just wondering how it is, as they get older, they tend to go through a stage where they’re not really open to empathy very much, are they? […] It seems like they can’t see that somebody’s angry. Sometimes they don’t recognize the subtleties of people being upset […]

### Teacher and school as key to success

Teachers highlighted the pivotal importance of being confident with the programme content in order to bring the content to life by, for example, experimenting with role play/drama rather than sticking too closely to the examples provided in the teaching materials. The potential for creativity and innovation was seen as a positive element of this programme, encouraging teachers to challenge their own professional development:

It makes you reflect on your own delivery and how it is coming across to the students and are they getting it, is there something that […] I’m not doing to enable interactive and probably what could I do better to make sure they understand and are getting the maximum from it that they can get […] I think it, it certainly developed me as a reflective practitioner in terms of my own teaching, which is not a bad thing.

Finally, the teachers suggested the embedding of the taught concepts and language not only in the curriculum but also within the larger ethos of the school. This would ensure that the learned concepts and the acquired common vocabulary could be revisited at transitional stages:

I think the way that this would be valuable is that it’s seen as this is something that we do throughout the school, and that maybe within year seven, some of the initial ideas are put down. […] a year later recap back on some of this […] what do you remember?

In summary, the three main themes identified through inductive thematic analysis indicate that the SPARK resilience programme has generally been experienced positively by teachers.

## Discussion

The current study aimed at empirically examining the treatment effects of the SPARK resilience programme, a new universal school-based intervention aimed at promoting resilience and preventing depression, featuring a novel but exploratory two cohort treatment/control design. Fulfilling both main objectives of the SPARK resilience programme, depression symptoms were significantly lower and resilience scores significantly higher in the treatment cohort compared to the control cohort, importantly, *after but not before* intervention. Effects were strongest and most persistent through all follow-up assessments for resilience whereas effects on depression waned over time so that differences between treatment and control group were no longer significant at the 12 month follow-up assessment.

The SPARK resilience programme performed particularly well regarding the promotion of resilience measured with a self-report questionnaire. Girls included in the treatment cohort reported higher resilience after treatment and even though resilience scores decreased at the 6 month follow-up assessment, they still remained significantly higher than then baseline scores and when compared to the control cohort. Furthermore, treatment effects were sustained through to the 12 month follow-up assessment suggesting that the intervention increased resilience at least up to 12 months after the programme ended. Regarding depression scores, the intervention led to a significant reduction of depression symptoms directly after the programme as well as 6 months later. However, at the 12 month follow-up assessment, depression scores returned to the baseline levels and also no longer differed from the control cohort, suggesting that preventative effects in regards to depression symptoms only lasted for about 6 months after the intervention ended. Nevertheless, the programme significantly reduced depression symptoms during the 6 months after the intervention and it remains to be determined whether these positive treatment effects could be sustained through the introduction of booster lessons one year after the intervention ended.

The positive intervention effects that emerged in the quantitative component of the study were reflected in the qualitative assessment. Teachers thought the intervention was helpful in providing tools and skills for the development of emotional resilience and for the identification of maladaptive thought patterns. The intervention was perceived as fostering empathy and better relationships between students and teachers, extending beyond the class room to the whole school climate.

The high risk status of the sample was evident in the high rates of girls scoring above the clinical cut-off score for depression (i.e. 7.7–12.0%) at all assessments of both treatment and control cohorts. However, the SPARK resilience programme proved effective in the promotion of mental health in this deprived community in spite of the high depression scores and even though the current intervention was administered by trained teachers in a universal setting rather than by professional psychologists in a targeted sample. The average effect size of the intervention regarding depression symptoms was relatively small with *d* = .24 but comparable to the average effect of a similar resilience intervention [14] and slightly higher compared to other preventative programmes [15–17]. The average effect size of SPARK regarding resilience scores was larger with *d* = .30.

The combination of a two cohort treatment/control design with a mixed methods approach turned out to be a useful and economical strategy for the preliminary evaluation of a universal intervention in an authentic school setting. Including two complete cohorts separated by one year ensured that there was no bias for inclusion to treatment or control group. Importantly, treatment and year-ahead control cohorts did not differ significantly on any psychometric measures before intervention, suggesting that the significant differences between post-treatment scores and control scores were a function of the intervention rather than of developmental change or initial group differences. Finally, growth curve analysis allowed for the investigation of change over time within the treatment cohort for each participant regardless of missing data. Hence, the chosen design proved useful for an initial investigation of the SPARK resilience programme’s efficacy, overcoming several of the challenges when evaluating school-based interventions (e.g., finding an adequate control group, nested structure of the data, attrition due to listwise deletion). In addition, the mixed method approach in the treatment cohort was not only helpful regarding the evaluation of the intervention but also provided important feedback for the further development of the SPARK resilience programme and its adaptation to specific schools. This is important, given that different schools serving different populations may require that the programme would be tailored to their specific needs in order to be maximally effective. Findings of the qualitative study also point to the importance of extending the intervention across the school years by introducing additional “booster” sessions, especially during more challenging periods. Such booster sessions, during which students will be reminded of the focal concepts of SPARK, may also increase and prolong the preventative but waning effects the intervention had on depression symptoms. Furthermore, the qualitative results also imply that the intervention had a more general positive impact on school climate and camaraderie among pupils. Future studies should investigate and control for the non-specific effects the intervention may have had on children and teachers across the school.

Though the current study has several strengths—outcomes reflecting both negative and positive aspects of psychological functioning, sample representing a high-risk population, adoption of an innovative mixed methods approach––it is important to highlight methodological limitations. Firstly, although our methodological approach proved effective in the preliminary testing of treatment efficacy, it does not represent a replacement for the gold standard of the randomized controlled trial. The main weakness of our applied approach was the single assessment of the control cohort. Even in the absence of significant differences between treatment cohort at the pre-treatment assessment and the control cohort at the 12-months follow-up assessment––as was the case in the current study––the possibility that the control cohort might have been significantly different from the treatment cohort at baseline, or that the control cohort changed over time, cannot be excluded. In other words, the applied study design does not allow testing the assumption whether the scores in the control group remained stable over time or not. In addition, there was a one year difference between treatment and control cohort. Consequently, while our two cohort treatment/control mixed methods design provides a promising exploratory approach for the preliminary testing of intervention efficacy, follow-up studies based on standard randomised controlled trials are still required in order to be able to draw causal conclusions about the efficacy of the intervention. Our methodological approach may be most suitable for gathering first empirical information on the efficacy of a school-based intervention. Resulting findings may then help deciding whether to conduct a standard randomized controlled trial that requires substantially more resources. A second limitation worth being noted is that demographic data as well as outcome measures were exclusively based on child self-report. In addition, depression and resilience was assessed with measures that have not been validated with children aged 11 years. Third, the sample included only girls. Fourth, the evaluation did not control for important covariates (e.g., socio-economic status of family, parenting quality, family structure, psychopathology of parents etc.). Fifth, there was no detailed assessment of intervention fidelity. Sixth, the outcome measures were not specifically developed for the use with adolescents. And, finally, qualitative results were based on a teacher focus group only rather than individual interviews with teacher and children.

### Conclusions

The current study provides preliminary evidence for the efficacy of a new universal school-based resilience-promoting intervention in a Western youth population most at risk for mental health problems. Exploratory findings suggest that the inclusion of a teacher-delivered resilience module into the standard curriculum proved effective in both decreasing depression and promoting resilience-related traits. While the positive treatment effects on depression symptoms dissipated after 6 months, the effects on resilience were sustained throughout the 12 months follow-up period. Hence, the current study further confirms that the broad implementation of resilience-promoting school programmes may be one effective and both practically and economically feasible approach to tackle the challenge of increasing adolescent mental health problems in the United Kingdom and other Western countries.

## Supporting information

S1 Dataset Data of the current paper is provided in the following SPSS file: MASTERFILE_ALL_DATA_27022017.sav. (SAV)
